# Artificial Anæsthesia as a Means of Facilitating Uterine Diagnosis

**Published:** 1855-04

**Authors:** 


Artificial Anæsthesia as a means of facilitating Uterine Diagnosis.—Since the first introduction of ether and chloroform into obstetric practice in 1847, Dr. Simpson has annually endeavored to point out in his lectures the great advantages that are occasionally derivable from their employment, in the way of facilitating obstetric diagnosis. The production of a state of anæsthesia has been found specially useful as a means of physical diagnosis, under the following circumstances:—

1. In cases of difficult parturition, the state of anæsthesia enables the accoucher to ascertain more easily and exactly, by his tactile examination, any peculiarities in the position or presentation of the child, or in the nature and amount of any impediment existing in the pelvic bones or maternal passages.

2. In instances of uterine or ovarian disease, connected with neuralgic tenderness of the abdominal walls and pelvis, it is often impossible to make a complete and useful tactile examination, unless the patient be previously anæsthized.

3. In spurious pregnancy, with its usual characteristic, abdominal distension, the use of chloroform at once, as is now well known, enables the practitioner to decide the nature of the case; the abdominal enlargement disappearing as the state of deep anæsthesia supervenes.

4. In the two preceding morbid states, and in any other cases of uterine or ovarian disease, requiring a very accurate tactile examination, the previous production of anæsthesia not only allows the tactile examination to be gone through without suffering, but further, it very greatly facilitates tne examination by the state of local and general relaxation which it induces. Under such relaxation, the physical examination of the uterine organs by touch is rendered infinitely more perfect.

5. In instances where a required examination is objected to, from motives of delicacy, the state of anæsthesia saves the feelings of the patient—a matter of no slight moment in the practice of the obstetric profession.—*Boston Med. & Surg Journal.*

